# Colovesical Fistula As An Uncommon Presentation Of Metastatic Lung Cancer

**DOI:** 10.7759/cureus.2767

**Published:** 2018-06-08

**Authors:** Thu-Cuc Nguyen, Aaron M Jaffe, David Gray, John Cain, Thomas Brown

**Affiliations:** 1 Internal Medicine, University of Central Florida College of Medicine, Orlando, USA; 2 Internal Medicine, University of Central Florida College of Medicine/Ocala Health, Orlando, USA; 3 Pathology, Ocala Health; 4 Radiology, University of Central Florida College of Medicine/Ocala Health, Orlando, USA

**Keywords:** colovesical fistula, lung adenocarcinoma, non-small cell lung cancer, faecaluria, pneumaturia, uti, metastatic lung adenocarcinoma

## Abstract

Colovesical fistula is an atypical communication between the colon and the bladder. The most common causes of colovesical fistula are diverticulitis, inflammatory bowel disease, lymphoma and complication from radiation therapy. Patients with colovesical fistula present with recurrent urinary tract infections (UTI), dysuria, frequency, abdominal pain, pneumaturia, faecaluria, and hematuria. We present a case of a patient with stage IV lung adenocarcinoma presented with abdominal pain, dysuria, and faecaluria who was found to have a colovesical fistula. Although colovesical fistula may be sequelae of advanced colon or bladder cancer, it is a very uncommon presentation of metastatic cancer from distant sites. Our case is the first to show that colovesical fistula may present from metastatic lung adenocarcinoma. Clinical awareness of this very unusual presentation of metastatic cancer can lead to faster diagnosis and treatment, possibly minimizing excessive use of antibiotics.

## Introduction

Colovesical fistula is an atypical communication between the lumen of the colon and the bladder. Most commonly, a colovesical fistula is formed as a result of diverticulitis which comprises approximately 60% of cases, colorectal cancer is responsible for around 20% of cases, inflammatory bowel diseases comprising nearly 10% of cases, less frequent etiologies include appendicitis, radiation therapy, and trauma [1]. Patients with colovesical fistula classically present with pneumaturia, fecaluria, recurrent urinary tract infections (UTI), dysuria, frequency, or passage of urine rectally. The fistulas most commonly will form a communication entering through the dome of the bladder representing approximately 60% of the cases. The posterior wall and trigone occur less frequently at around 30% and 10% respectively [1].

Non-small cell lung carcinoma (NSLC) is the most common form of lung cancer, and is broken down into 3 subtypes including adenocarcinoma, squamous cell carcinoma, and large cell carcinoma. Approximately 50% of all newly diagnosed lung cancers have distant metastasis at presentation [2]. The most common sites of metastatic disease in patients with lung cancer include brain, liver, adrenal glands, and bones [3-4] .

## Case presentation

Patient is a 79 year-old man with history of hypertension and coronary artery disease initially presented for evaluation of a palpable right neck mass with associated symptoms of dyspnea on exertion and 36lb weight loss. Workup revealed stage 4 adenocarcinoma with negative anaplastic lymphoma kinase (ALK), c-ros oncogene 1 (ROS1), epidermal growth factor mutation (EGFR) and positive for programmed death-ligand 1 (PD-L1). Positron emission tomography/computed tomography (PET/CT) revealed disease mostly restricted to the thoracic area, but there was also dense hypermetabolic activity with mural thickening in the sigmoid colon of unclear significance. Patient refused nivolumab systemic therapy and was treated with palliative radiation therapy to his right neck mass, right hilum and mediastinum with good palliative response.

**Figure 1:**
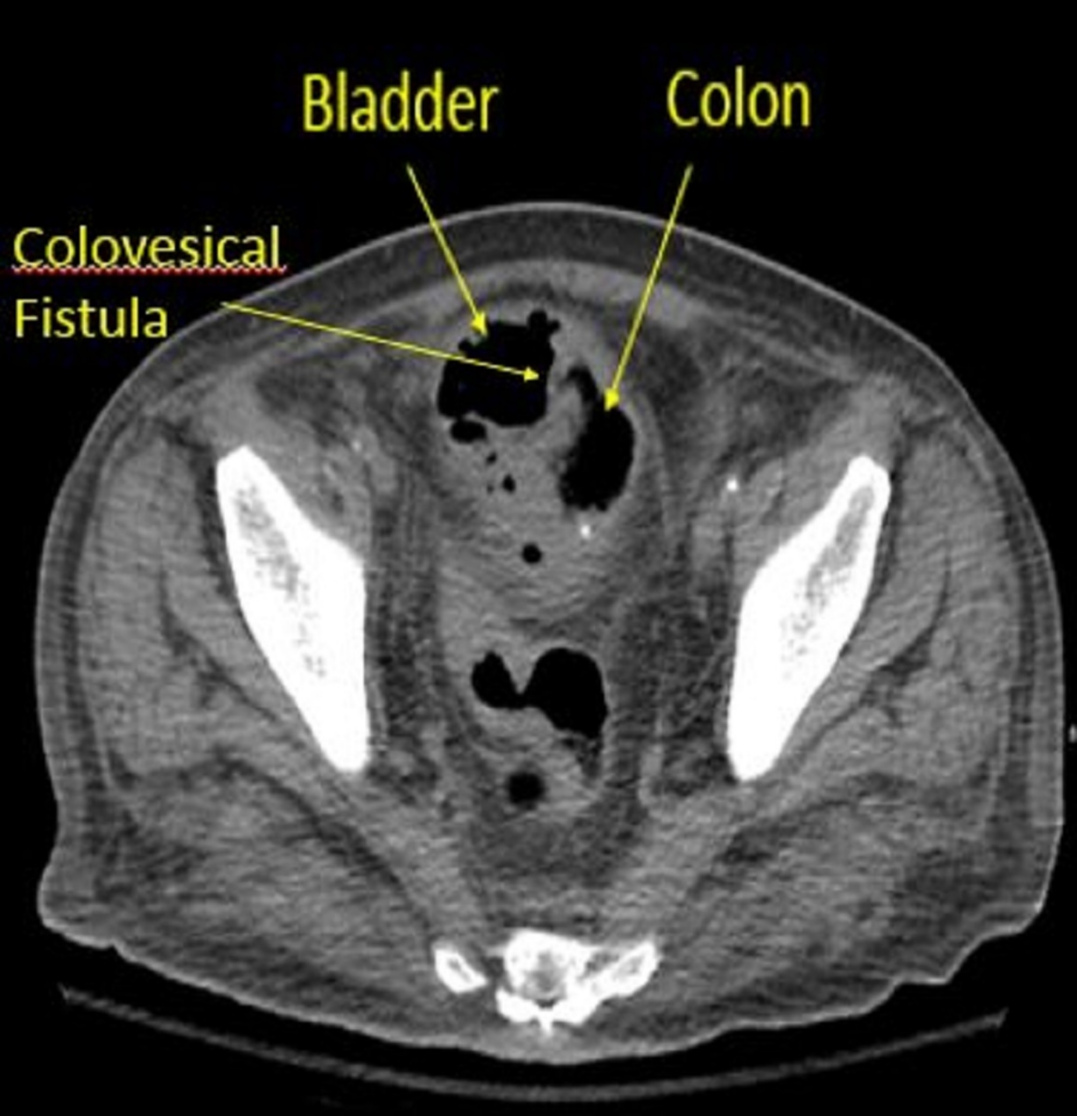
Computed tomography abdomen and pelvis scan showing colovesical fistula forming between colon and bladder with air in the urinary bladder.

Two weeks after radiation therapy, patient presented to the hospital with dysuria, fecaluria and abdominal pain which did not improve after taking a course of antibiotics. CT of the abdomen and pelvis revealed a colovesical fistula between the sigmoid colon and bladder, large mesenteric lymph node just above the sigmoid colon mass and wall thickening in the sigmoid colon (Figure 1). 

Cystoscopy revealed edematous mucosa and exudation of feculent material from the fistula site. Sigmoidoscopy revealed a fistula in the distal to mid sigmoid colon about 25 cm from dentate line and a 3 cm area of extrinsic compression.

**Figure 2:**
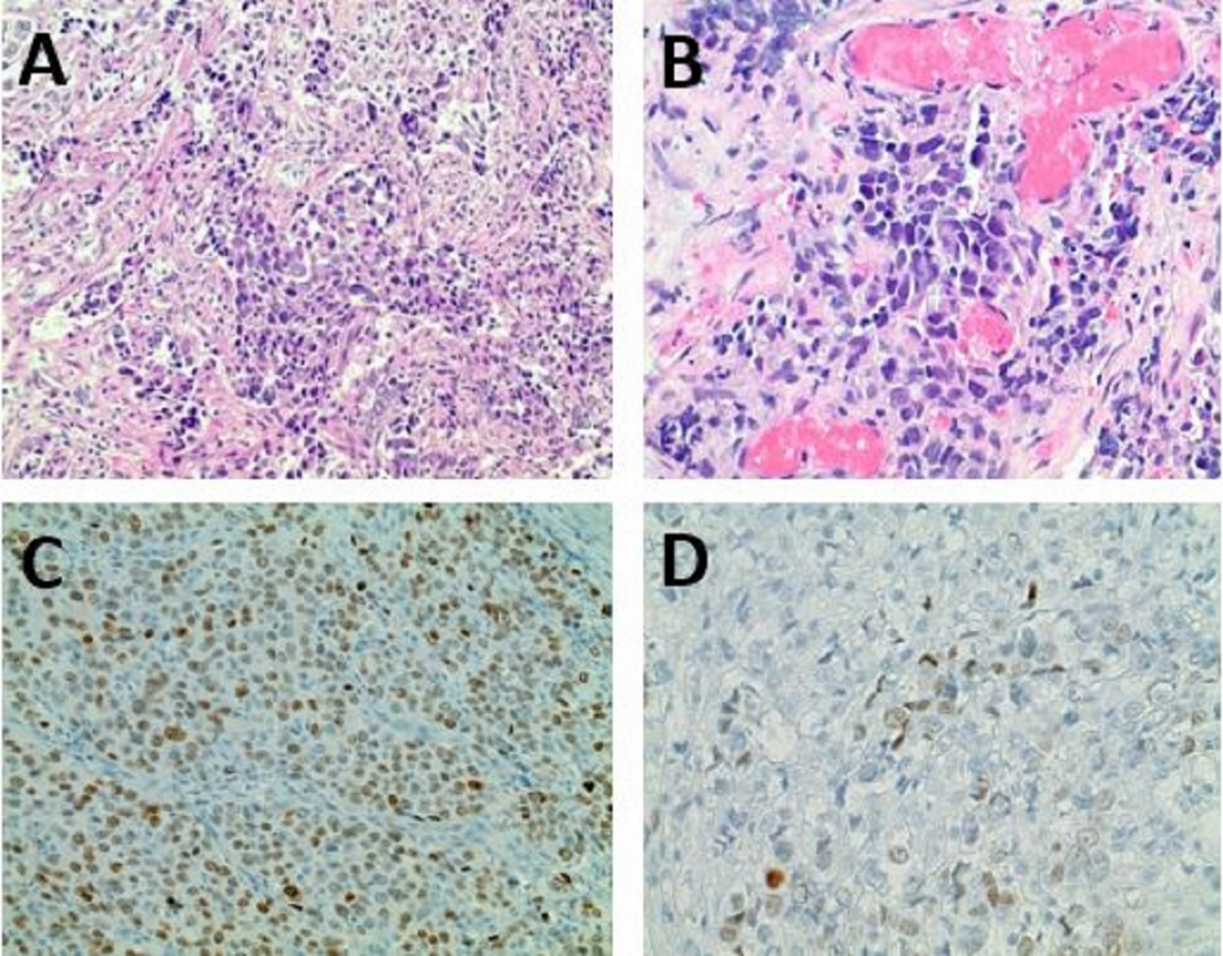
Haemotoxylin and eosin staining from (A) colovesical fistula mass and (B) primary lung cancer mass; Thyroid transcription factor-1 (TTF-1) staining for (C) colovesical fistula mass and (D) primary lung cancer mass

Patient was taken to surgery for resection of colovesical fistula mass, sigmoidectomy with colostomy, drainage of the intraabdominal abscess and repair of a complicated bladder fistula. Pathology of the tumor revealed metastatic poorly differentiated lung adenocarcinoma with an associated colovesical fistula and extensive lymphovascular invasion. Comparison of resected colovesical fistula mass to primary lung cancer biopsy showed similar morphology by Haemotoxylin and Eosin staining (H&E) and lung cancer specific thyroid transcription factor-1 (TTF1) marker consistent with the colovesical fistula mass being metastatic lung cancer (Figures 2A-D). 

## Discussion

Our case is the first in the literature to show that colovesical fistula can develop from a lung cancer metastasis. Our patient did not have evidence of diverticulitis or inflammatory bowel disease on colonoscopy or pathological evaluation, ruling out these common causes of colovesical fistulas. He does not have any history of radiation to the abdomen or pelvis.

The most common symptoms of colovesical fistula are urinary urgency, dysuria, tenesmus, suprapubic pain, pneumaturia, fecaluria, hematuria, and UTI. Colovesical fistula is more common in males than in females since the uterus and the broad ligaments serve as a barrier between sigmoid colon and the bladder [5]. Clinical history, radiographic imaging, colonoscopy, and cystoscopy are essential in diagnosing colovesical fistula. Urinalysis usually shows leukocytes, bacteria, and debris. Urine culture usually grows mixed fecal flora, although Escherichia coli is usually the predominant organism [6]. Computed tomography (CT) scan is the most sensitive test to diagnose colovesical fistula. CT scanning of the abdomen and pelvis demonstrate small amounts of air or contrast material in the bladder, localized thickening of the bladder wall, or an extraluminal gas-containing mass adjacent to the bladder [5,6]​​​​​. Cystoscopy is an essential diagnostic procedure. It can be used to rule out malignancy; however, it fails to visualize the opening. Colonoscopy is helpful in determining the nature of the bowel disease, but it is not accurate in detecting the fistula [6]. Our patient is proven to have colovesical fistula based on clinical presentation, colonoscopy, CT abdomen and pelvis, and pathology evaluation of the tumor mass.

Management of colovesical fistula can be divided into conservative management and surgical management. Conservative management is reserved for patients with multiple comorbidities or elderly who cannot tolerate general anesthesia. Patients can be managed with medical therapy with periodic administration of antibiotics and supportive care with urethral catheter drainage [5]. For surgical management, primary resection of the colon with anastomosis performed as a one-stage procedure is the standard treatment of colovesical fistula when the cause is diverticular or granulomatous bowel disease [7]. Staged repairs may be preserved for patients with large intervening pelvic abscesses or those with advanced malignancy [7]. Our patient underwent palliative surgery to manage complications from colovesical fistula.

## Conclusions

Our case is the first in the literature to show that colovesical fistula can develop from a lung cancer metastasis. Clinical awareness of this very unusual presentation of metastatic cancer can lead to faster diagnosis and treatment, possibly minimizing excessive use of antibiotics.

## References

[1] Pollard SG, Macfarlane R, Greatorex R, Everett WG, Hartfall WG (1987). Colovesical fistula. Ann R Coll Surg Engl.

[2] Yang CJ, Hwang JJ, Kang WY, Chong IW (2006). Gastro-intestinal metastasis of primary lung carcinoma: Clinical presentations and outcome. Lung Cancer.

[3] Salah S, Tanvetyanon T, Abbasi S (2012). Metastatectomy for extra-cranial extra-adrenal non-small cell lung cancer solitary metastases: systematic review and analysis of reported cases. Lung Cancer.

[4] Hillers TK, Sauve MD, Guyatt GH (1994). Analysis of published studies on the detection of extrathoracic metastases in patients presumed to have operable non-small cell lung cancer. Thorax.

[5] Scozzari G, Arezzo A, Morino M (2010). Enterovesical fistulas: diagnosis and management. Tech Coloproctol.

[6] Pindoria K, Reyad A, Youssef Youssef, Y Y (2014). Foley catheter through a colovesical fistula: an unusual method of diagnosis. BMJ Case Reports.

[7] McBeath RB, Schiff M Jr, Allen V, Bottaccini MR (1994). 12 year experience with enterovesical fistulas. Urology.

